# The OMNIVEG Study: Effects of Transitioning from a Traditional to a Vegan Mediterranean Diet on Fat Oxidation During Exercise

**DOI:** 10.3390/nu17142274

**Published:** 2025-07-09

**Authors:** Miguel López-Moreno, Ujué Fresán, Juan Del Coso, Alejandro Muñoz, Millán Aguilar-Navarro, María Teresa Iglesias-López, Francisco J. Amaro-Gahete, Jorge Gutiérrez-Hellín

**Affiliations:** 1 Diet, Planetary Health and Performance, Faculty of Health Sciences, Universidad Francisco de Vitoria, 28223 Madrid, Spain; miguel.lopez@ufv.es; 2 Food Biotechnology, Universidad Francisco de Vitoria, 28223 Madrid, Spain; 3 ISGlobal, 08024 Barcelona, Spain; ujue.fresan@isglobal.com; 4 Sport Sciences Research Centre, Rey Juan Carlos University, 28943 Madrid, Spain; 5 Exercise and Sport Science, Faculty of Health Sciences, Universidad Francisco de Vitoria, 28223 Madrid, Spain; alejandro.munoz@ufv.es (A.M.); millan.aguilar@ufv.es (M.A.-N.); 6 Wellness and Health, Universidad Francisco de Vitoria, 28223 Madrid, Spain; m.iglesias.prof@ufv.es; 7 Department of Physiology, Faculty of Medicine, Sport and Health University Research Institute (iMUDS), University of Granada, 18016 Granada, Spain; amarof@ugr.es; 8 Centro de Investigación Biomédica en Red Fisiopatología de la Obesidad y Nutrición (CIBERobn), Instituto de Salud Carlos III, 28029 Madrid, Spain; 9 Instituto de Investigación Biosanitaria de Granada (Ibs.GRANADA), 18014 Granada, Spain

**Keywords:** substrate oxidation, vegan diet, maximal oxidation rate, carbohydrate oxidation rate, vegans, diet therapy

## Abstract

Background: This study aimed to evaluate the changes in fat utilization associated with transitioning from a traditional to a vegan Mediterranean diet in healthy, physically active men during a ramp exercise test. Methods: In a controlled crossover design, fourteen healthy, physically active men followed a traditional Mediterranean diet for three weeks (baseline). Then, participants transitioned to a four-week isocaloric vegan version of the Mediterranean diet, matched for macronutrient distribution but excluding all animal foods. Immediately after each dietary intervention, participants completed an incremental exercise test (from 30% to 70% of VO_2peak_) on a cycle ergometer in a fasted state to determine peak fat oxidation (PFO) and its associated exercise intensity (Fatmax). Exercise heart rate and the rating of perceived exertion were also recorded at each exercise intensity. Results: The traditional and vegan Mediterranean diets provided comparable amounts of energy (2599.6 ± 180.8 and 2634.9 ± 148.3 kcal/day, *p* = 0.140) and total fat (97.0 ± 17.8 and 99.0 ± 13.2 g/day; *p* = 0.620). However, the vegan Mediterranean diet contained a lower proportion of saturated fat (25.2 ± 6.8 vs. 13.6 ± 4.4% of total fat, *p* < 0.010). Still, the dietary transition was not associated with modifications in PFO (0.323 ± 0.153 and 0.347 ± 0.147 g/min; *p* = 0.678) or Fatmax (40.51 ± 7.30 and 40.51 ± 10.71%VO_2peak_; *p* = 1.000) during exercise. Moreover, the dietary transition did not significantly change the response curves across exercise intensities for fat oxidation (*p* = 0.553), heart rate (*p* = 0.280), or the rating of perceived exertion (*p* = 0.433). Conclusions: Switching from a traditional to a vegan Mediterranean diet did not affect fat oxidation, exercise intensity at peak fat oxidation, or perceptual responses during exercise in healthy, active men. These findings suggest that physically active individuals can adopt a vegan version of the Mediterranean diet without compromising fat utilization during submaximal aerobic exercise. Clinical Trial Registry: NCT06008886. Date of registration: 28 July 2023.

## 1. Introduction

The Mediterranean diet is a traditional dietary pattern originating from countries surrounding the Mediterranean Sea [1,2]. It is predominantly plant-based, featuring high consumption of vegetables, fruits, legumes, whole grains, and nuts, with olive oil serving as the principal source of fat [3] due to its ubiquitous use in meal preparation [4,5]. Animal products are included in moderation within the traditional Mediterranean diet, with fish and poultry consumed more regularly than red meat and low-to-moderate amounts of dairy products like cheese or milk [6,7]. This dietary approach is also marked by a low intake of processed foods and added sugars and often includes a modest amount of red wine consumed with meals [8,9]. The Mediterranean diet provides a well-balanced distribution of macronutrients and a high density of micronutrients, dietary fiber, and bioactive compounds with antioxidant and anti-inflammatory properties [6]. The core components of the Mediterranean diet are also commonly found in the diets of the five Blue Zones around the world, suggesting that these shared elements may be key to their associated health benefits [10]. Overall, there is ample evidence supporting the benefits of the Mediterranean diet for metabolic regulation, vascular integrity, and a reduced risk of chronic diseases [11]. The Mediterranean diet also slows down the progression of aging and helps to prevent the onset of frailty [12]. Indeed, it is widely acknowledged as a reference dietary pattern for maintaining overall health in the general population, as well as a therapeutic strategy in the prevention and management of cardiovascular diseases [13,14]. Moreover, its nutritional composition suggests potential benefits for physically active individuals and athletes, particularly in supporting recovery, reducing exercise-induced oxidative stress, and enhancing long-term performance [15,16].

In recent years, the Mediterranean diet has been proposed as a potential nutritional model for athletes [15]. The food composition of the Mediterranean diet results in a high intake of bioactive compounds, such as polyphenols, omega-3 fatty acids, and dietary nitrates, which have been associated with reduced systemic inflammation and oxidative stress, two key physiological stressors induced by intense physical exercise [17]. Additionally, it has been found that the Mediterranean diet may offer ergogenic benefits when adopted by athletes transitioning from less healthy or Western-style dietary patterns [18]. It is important to note that much of the evidence supporting these effects comes from studies conducted in non-athlete populations; therefore, their applicability to athletic performance remains largely theoretical and requires further investigation [15]. Moreover, the traditional macronutrient profile of the Mediterranean diet is moderate in carbohydrate content, which may be insufficient for athletes with high glycogen requirements, particularly those involved in endurance or high-intensity sports [17]. In the same vein, while protein needs can potentially be met through sources such as fish, legumes, and dairy within the Mediterranean diet, targeted adjustments in timing or supplementation may be necessary to support muscle maintenance and recovery [19,20]. Last, the inclusion of fish and other animal-based foods as primary sources of high-quality protein and long-chain omega-3 fatty acids may limit the suitability of the Mediterranean diet for vegetarian or vegan athletes [21], who may need to adapt or substitute key components to meet their nutritional requirements without compromising the diet’s potential benefits.

Incorporating legumes, soy products, nuts, seeds, and fortified foods—while eliminating animal-based fats and proteins of the Mediterranean diet—can help vegetarian and vegan athletes meet their nutritional needs. This approach ensures adequate intake of protein, iron, vitamin B12, and omega-3 fatty acids while maintaining the Mediterranean diet’s emphasis on whole, minimally processed, nutrient-dense foods [22]. This concept has been referred to as the vegan Mediterranean diet [23], a potential alternative dietary pattern that retains the core principles of the Mediterranean diet while excluding all animal-derived products. In the vegan Mediterranean diet, animal fats and proteins are substituted by increases in protein-rich plant-based foods and a higher amount of vegetable fats, which is typically accompanied by a reduction in the proportion of saturated fats. As the vegan version of the Mediterranean diet has only vegetal sources of protein and fat, it may offer additional advantages, including reductions in blood pressure and concentrations of cholesterol in physically active individuals [23] and a more environmentally sustainable pattern [24]. However, evidence of the benefits of the vegan Mediterranean diet is scarce, especially when compared to the traditional version, which is already considered a healthy dietary pattern. In this context, controlled trials are needed to provide a more comprehensive understanding of the comparative health effects of these dietary patterns in clinical populations and in athletes.

For instance, it is well established that dietary fat intake can influence substrate oxidation during exercise, with diets with higher fat intake promoting a greater reliance on fat as a fuel source while reducing carbohydrate utilization at a given exercise intensity [25,26]. While the traditional Mediterranean diet includes both saturated and unsaturated fat sources, the vegan adaptation of this dietary pattern is predominantly composed of unsaturated fats, primarily derived from nuts, seeds, and plant oils. In this context, increasing dietary intake of unsaturated fats—particularly monounsaturated and polyunsaturated fatty acids—may be associated with higher fat oxidation capacity during exercise; a factor potentially beneficial for endurance performance [27]. Moreover, the formation of short-chain fatty acids derived from dietary fiber intake may enhance fatty acid β-oxidation and improve insulin sensitivity [28]. However, to the best of the authors’ knowledge, no study to date has compared substrate oxidation during exercise between the traditional and vegan versions of the Mediterranean diet. Therefore, this study aimed to evaluate the changes in fat utilization associated with transitioning from a traditional to a vegan Mediterranean diet in healthy, physically active men during a ramp exercise test. We hypothesized that adopting a vegan Mediterranean diet would be associated with higher fat oxidation rates across various exercise intensities.

## 2. Materials and Methods

### 2.1. Participants

From an initial sample of seventeen participants, only fourteen finalized the whole experiment, including diet interventions and measurements. Three participants withdrew during the experiment for different reasons: One during the traditional Mediterranean diet due to adherence challenges, and two during the vegan phase, one for time constraints and another for unrelated mild health issues. The final sample included fourteen participants who completed both dietary interventions and were included in the analysis. They had a mean (standard deviation) age of 24.6 (7.0) years, body mass of 74.9 (8.9) kg, body mass index of 23.5 (1.6) kg/m^2^, and 43.7 (10.1) mL/kg/min of peak oxygen uptake (VO_2peak_). Inclusion criteria required participants to (a) be male and aged between 18 and 40 years following a traditional Mediterranean diet; (b) engage in moderate-intensity exercise 3–5 days per week; (c) perform at least 60 min of exercise per session; (d) have a body mass index (BMI) between 18.5 and 24.9 kg/m^2^; (e) be non-smokers; (f) consume minimal to no alcohol; and (g) have no orthopedic impairments. Exclusion criteria included (a) a history of chronic cardiovascular, metabolic, gastrointestinal, respiratory, or musculoskeletal diseases and (b) any significant musculoskeletal injuries within the six months preceding the study. Participants were recruited through the distribution of flyers across the campus of Universidad Francisco de Vitoria in Madrid. Participants were recruited between September and December 2023. Eligibility was assessed using a pre-participation questionnaire and an initial screening process, which collected detailed information on participants’ medical history, diet patterns, and physical activity levels. As part of this screening, a registered dietitian verified that participants habitually followed the core principles of the traditional Mediterranean diet, including high intake of plant-based foods and moderate consumption of fish, poultry, and dairy products. Prior to enrollment, all potential participants were fully informed about the possible risks and discomforts associated with the study. Written informed consent was obtained from each individual, confirming their voluntary participation. The study protocol was reviewed and approved by the Ethics Committee of the University Francisco de Vitoria (approval number: 20/2023; approval date: 12 June 2023) and complied with the ethical principles of the Declaration of Helsinki. The trial was prospectively registered on ClinicalTrials.gov (identifier: NCT06008886; date of registration: 28 July 2023).

### 2.2. Study Design

**Figure 1:**
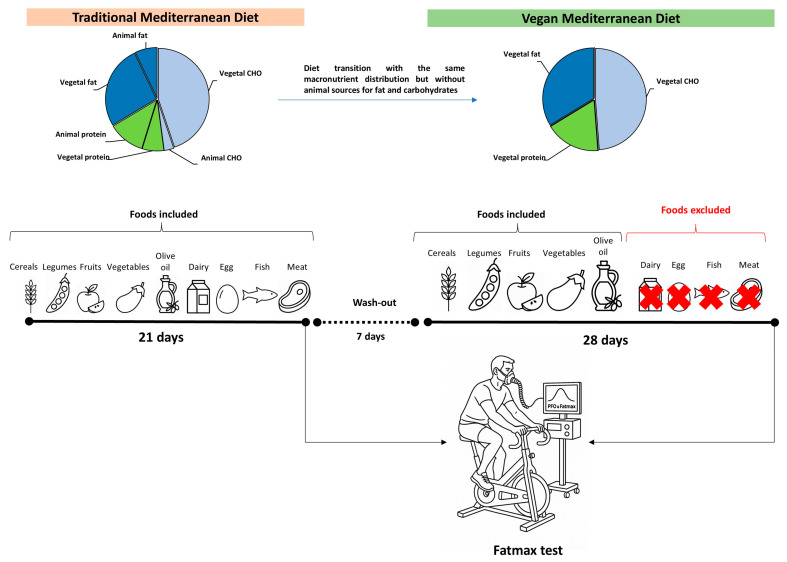
Experimental design of a dietary intervention study comparing a traditional Mediterranean diet with a vegan Mediterranean diet. The study consisted of three phases: (1) A 21 days on a traditional Mediterranean diet including cereals, legumes, fruits, vegetables, olive oil, dairy products, eggs, fish, and meat; (2) a 7-day wash-out period; and (3) 28 days on a vegan Mediterranean diet excluding all animal-derived products. Macronutrient distribution is illustrated for both diets, highlighting the shift from mixed animal and vegetal sources to exclusively vegetal sources without affecting macronutrient distribution. A Fatmax test was conducted to assess metabolic responses during exercise at the end of each dietary intervention.

This controlled crossover trial was derived from the OMNIVEG study [23]. The OMNIVEG study involved two distinct dietary phases: Participants initially completed a 3-week baseline period following the general principles of a traditional Mediterranean diet, after which they transitioned to a 4-week intervention phase involving an isocaloric vegan adaptation of the Mediterranean diet. A seven-day washout interval separated the two phases to reduce potential carryover effects. Throughout the study, participants were instructed to maintain their usual lifestyle and physical activity levels, which were monitored through training logs to ensure consistency, while only their diet was modified (Figure 1). Both dietary protocols (i.e., traditional and vegan) were customized to align with individual food preferences while maintaining consistent macronutrient distributions across phases. The primary dietary modification in the vegan phase involved replacing animal-based sources of protein and fat with plant-based alternatives that matched the original macronutrient profile. As a result, animal-derived carbohydrates (e.g., lactose from dairy products), proteins (e.g., fish, meat, and poultry), and fats (e.g., from fish, meat, and poultry) constituted a low-to-moderate proportion of the total macronutrient content in the traditional Mediterranean diet but were absent in the vegan Mediterranean diet (Table 1).

### 2.3. Transition from a Traditional to a Vegan Mediterranean Diet

Prior to the start of the study, participants attended a pre-experimental session during which a registered dietitian conducted a comprehensive dietary assessment. This session included verification of adherence to a traditional Mediterranean dietary pattern—characterized by a high intake of vegetables, fruits, legumes, whole grains, and moderate consumption of animal products. Participants’ food preferences were recorded, and daily energy requirements were estimated. Using this information, the dietitian developed a normocaloric Mediterranean meal plan designed to align closely with each participant’s habitual eating patterns. The traditional Mediterranean diet was composed of modest amounts of fish, poultry, low-fat dairy, and eggs, with minimal red and processed meats, and excluded sweets. Olive oil served as the primary added fat, and animal fat only represented 21% of the total amount of fat. Animal sources contributed 62.9% of the total protein intake (Table 1). Participants followed this individualized traditional Mediterranean diet for a period of three weeks. After this phase, a one-week washout period was implemented, during which participants returned to their pre-experimental dietary routines. In practice, these routines were very similar to the traditional Mediterranean diet, as participants had been recruited based on their habitual adherence to this dietary pattern. The main purpose of the washout week was not to change dietary exposure but rather to provide a short recovery period between interventions and to allow participants to feel temporarily unmonitored, which may help reduce psychological fatigue and improve compliance in the subsequent phase. After the one-week washout phase, participants transitioned to a vegan, isocaloric version of the Mediterranean diet for four weeks. The vegan diet maintained the same macronutrient composition and included many of the same foods, but animal-derived protein and fat sources were substituted with plant-based alternatives providing comparable nutrient content. For example, fish, meat, and poultry were substituted with tofu, tempeh, and seitan; dairy products were replaced by fortified plant-based drinks and yogurts; and eggs were removed in favor of legumes, soy products, and nuts. Olive oil remained the principal fat source. As a result, the vegan version of the diet contained no animal-derived carbohydrates, fats, or proteins. To prevent any vitamin B12 deficiency during the vegan phase, participants received 1000 µg of cyanocobalamin twice per week. To facilitate adherence to both the traditional and vegan Mediterranean diets, participants received individualized meal plans with detailed instructions, including recommended food items, portion sizes, and brand suggestions, and were instructed to follow these plans consistently throughout each dietary phase. Compliance with the dietary protocols was monitored through three 24 h dietary recalls per week, and nutrient intake was analyzed using Dietopro software (Valencia, Spain) to ensure adherence throughout both diet phases. Additionally, participants received regular follow-up from the research team to address any dietary challenges throughout the study. Since the aim of this study was to examine the transition from a more widely adopted diet (i.e., the Mediterranean diet) to a less common and less scientifically established version (i.e., the vegan Mediterranean diet), participants were not randomly assigned to dietary interventions. Moreover, neither participants nor experimenters were blinded to the dietary assignments, as blinding was not feasible in this context.

### 2.4. VO_2peak_ and Exercise Intensity Standardization

One day after completing both the baseline period with the traditional Mediterranean diet and the intervention with the vegan Mediterranean diet, participants underwent a VO_2peak_ test. On this day, they performed a maximal cycling test to volitional fatigue. The test was preceded by a standardized warm-up consisting of 10 min at 50 W on a cycle ergometer (Ergoselect 4, Ergoline, Bitz, Germany). Following the warm-up, the workload increased by 25 W every minute, as previously described [30]. Participants were instructed to maintain a cadence between 70 and 90 rpm throughout the test. The test was terminated when participants were unable to sustain a cadence above 50 rpm or chose to stop pedaling due to fatigue. During the test, oxygen uptake (VO_2_) and carbon dioxide production (VCO_2_) were measured breath-by-breath using a gas analyzer (Ergostik, Geratherm Respiratory, Bad Kissingen, Germany). VO_2peak_ was defined as the highest VO_2_ value recorded during the test. To normalize exercise intensity for the subsequent Fatmax test (workloads eliciting 30–70% of each participant’s VO_2peak_), a regression analysis was performed for each individual, relating workload (in W) to VO_2_ (in L/min) based on data from the VO_2peak_ test. During the VO_2peak_ test, participants also adjusted and fixed the saddle and handlebar positions on the cycle ergometer and wore the same clothing they would use in the Fatmax test.

### 2.5. Peak Fat Oxidation Rate (PFO) and Fatmax

Two days after completing the VO_2peak_ test, conducted either after the baseline period with the traditional Mediterranean diet or after the vegan Mediterranean diet, participants performed a Fatmax test to obtain PFO and the exercise that elicited this PFO. Participants were encouraged to maintain their assigned dietary pattern (traditional or vegan Mediterranean diet) until the completion of the Fatmax test. Adherence during this post-intervention period was verified through additional 24 h dietary recalls. For this measurement, participants performed a 10 min standardized warm-up at a workload equivalent to 30% of VO_2peak_ (as measured in the previous test) on the cycle ergometer. Exercise intensity was then increased by 10% of VO_2peak_ every 3 min until reaching a workload equivalent to 70% of VO_2peak_. This upper limit was chosen because the respiratory exchange ratio exceeded 1.0 at intensities beyond this one, and therefore, the calculation of substrate oxidation was unfeasible. The day before the Fatmax trial, participants completed a light, standardized training session and were instructed to avoid alcohol, caffeine, and *p*-synephrine. They were also required to maintain a regular sleep pattern, ensuring at least 8 h of sleep [31,32]. Prior to each trial, the gas analyzer and flowmeter were calibrated using certified calibration gases (16.0% O_2_; 5.0% CO_2_, Sanro, Madrid, Spain) and a 3-L syringe.

On the day of the experimental trials, participants arrived at the laboratory at 08:00 a.m. in a fasted state (having not eaten for at least 8 h). Upon arrival, they provided a urine sample to confirm euhydration status, verified by urine-specific gravity of <1.020 [33]. Once compliance with all standardization procedures was confirmed, participants wore cycling shorts and a heart rate monitor (H10, Polar, Kempele, Finland). They then performed the Fatmax test on the same cycle ergometer used during the VO_2peak_ test. At the end of each workload, the rating of perceived exertion was measured with the 6–20-point Borg scale [34]. Participants were instructed to maintain a cycle cadence of 70 to 90 rpm during the whole test, which was noted in the Fatmax trial (after the traditional Mediterranean diet) and was replicated in the second Fatmax trial (after the traditional Mediterranean diet). During the whole exercise trial, expired gases were collected and measured by the same breath-by-breath analyzer used for the pre-experimental trial, and representative values of VO_2_ and VCO_2_ and heart rate were assessed for each workload by averaging the last 60 s of each stage. Participants were instructed to maintain a stable seated position on the cycle ergometer during the final 60 s of each stage, without lifting their buttocks from the saddle. The rate of energy expenditure and fat and carbohydrate oxidation at each stage were calculated from stoichiometric equations assuming that urinary nitrogen excretion was negligible [35,36]. Energy expenditure (kcal/min) during exercise was calculated as (3.869 × VO_2_) + (1.195 × VCO_2_), where VO_2_ and VCO_2_ are in L/min. Fat oxidation rate (g/min) was calculated as (1.67 × VO_2_) − (1.67 × VCO_2_), and carbohydrate oxidation rate (g/min) was calculated as (4.55 × VCO_2_) − (3.21 × VO_2_). In each Fatmax test, PFO (in g/min) was individually calculated for each participant as the highest value of fat oxidation rate obtained during the incremental exercise intensity test. The exercise intensity at which PFO was obtained for each individual was categorized as Fatmax, expressed as %VO_2peak_. There were no changes to trial outcomes after the trial commenced.

### 2.6. Statistical Analysis

Normal distribution of the dataset for each variable was confirmed using a Shapiro-Wilk test, and parametric statistics were employed as all variables presented a normal distribution. Student’s *t*-test for paired samples was used to compare dietary intake data obtained with the recalls during the traditional and vegan versions of the Mediterranean diet. A one-way analysis of variance (ANOVA) was used to compare PFO and Fatmax after each diet. A two-way ANOVA (diet × exercise intensity; 2 × 5) was used to compare energy expenditure, fat and carbohydrate oxidation rates, heart rate, and perceived exertion during exercise. The sphericity assumption was checked with Mauchly’s test. In the case of a main effect of diet, exercise intensity, or an interaction between these two factors, pairwise comparisons between the diets (i.e., data at the same exercise intensity with each diet) were identified with LSD posthoc tests. The effect size was assessed with the Cohen’s *d* in those cases with a statistically significant difference between diets. The following criteria were established to assess the magnitude of the effect: trivial (0–0.19), small (0.20–0.49), medium (0.50–0.79), and large (≥0.80). The significance level was set at *p* ≤ 0.050. The statistical analysis was conducted using the IBM Statistical Package for Social Sciences (SPSS) version 28.0 (IBM, Chicago, IL, USA).

## 3. Results

**Figure 2:**
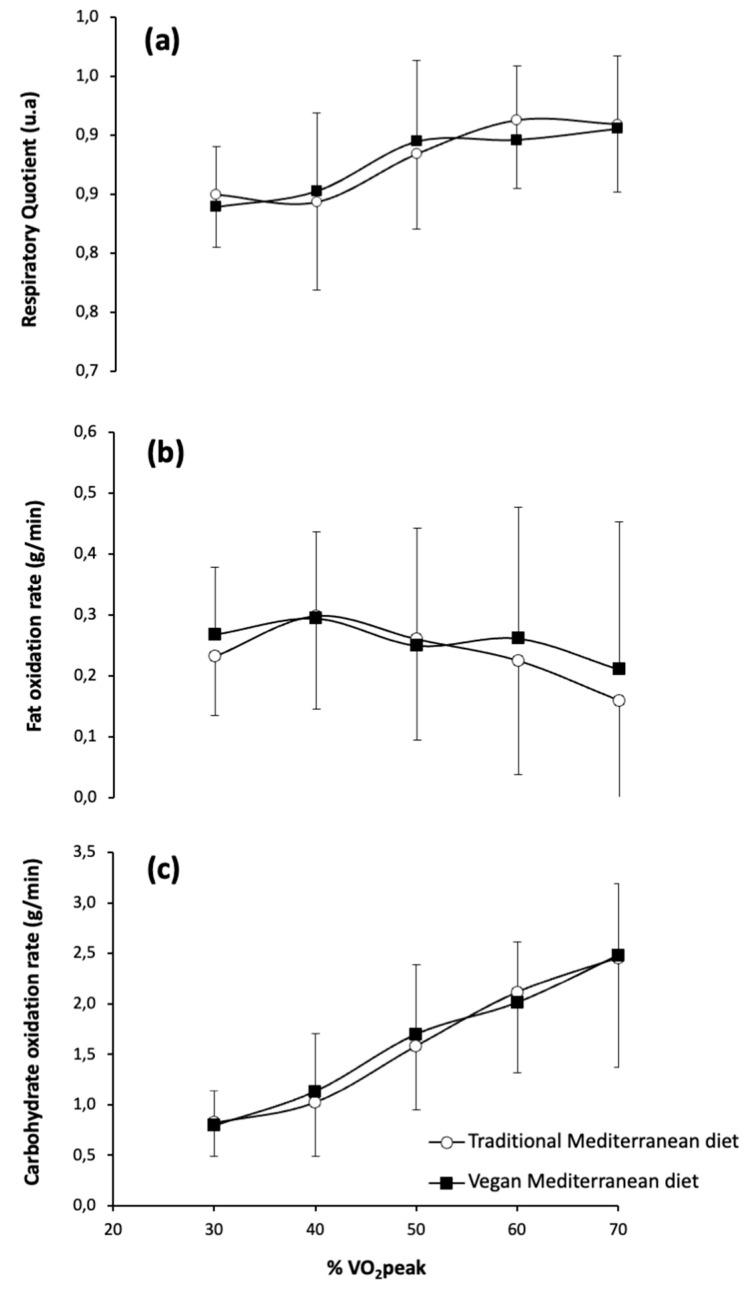
Respiratory exchange ratio (**a**), fat oxidation rate (**b**), and carbohydrate oxidation rate (**c**) during exercise of increasing intensity after the baseline period with the traditional Mediterranean diet and after the period with the vegan version of the Mediterranean diet. Data are mean ± SD for 14 participants.

Figure 2 shows the respiratory exchange ratio and fat and carbohydrate oxidation rates during exercise at increasing intensities after the traditional Mediterranean diet versus the vegan Mediterranean diet. For the respiratory exchange ratio, there were no significant main effects observed for diet (*F* = 0.074; *p* = 0.795), exercise intensity (*F* = 1.834; *p* = 0.224), or the interaction between diet and exercise intensity (*F* = 2.558; *p* = 0.161). Likewise, no significant main effects were observed for diet (*F* = 0.419; *p* = 0.553), exercise intensity (*F* = 3.182; *p* = 0.248), or the interaction between diet and exercise intensity (*F* = 1.647; *p* = 0.399) on the fat oxidation rate during exercise. For carbohydrate oxidation rates during exercise, there were no significant main effects for diet (*F* = 3.853; *p* = 0.107) and the diet × intensity interaction (*F* = 1.023; *p* = 0.493), but a significant main effect of exercise intensity was observed (*F* = 226.402; *p* < 0.001), indicating that carbohydrate oxidation increased with exercise intensity regardless of diet. Despite this significant main effect on carbohydrate oxidation rate, the posthoc analysis did not reveal any differences between the traditional and vegan versions of the Mediterranean diet at any exercise intensity.

**Figure 3:**
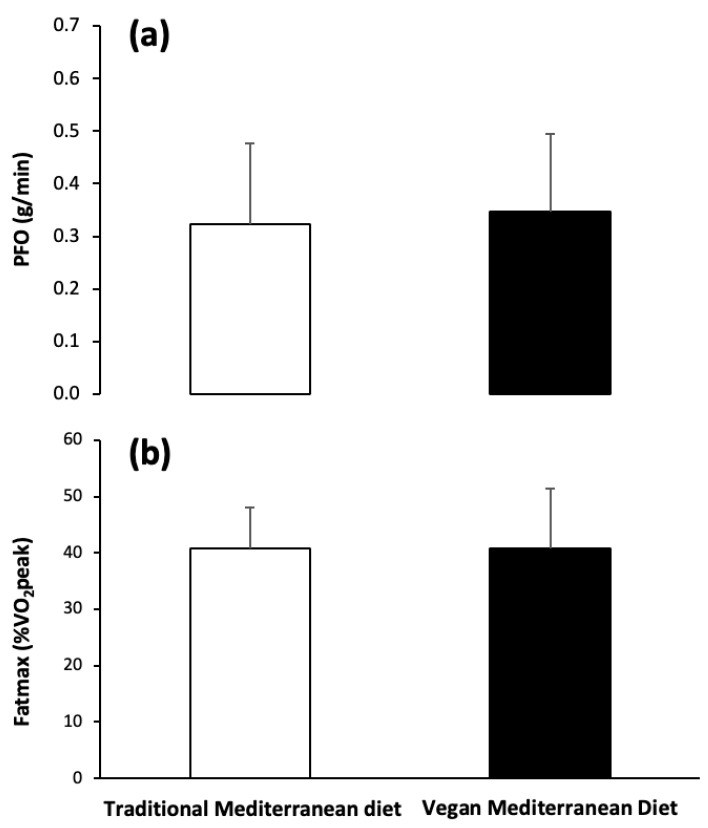
Peak fat oxidation rate (PFO; (**a**)) and exercise intensity at which PFO is achieved (Fatmax; (**b**)) during exercise of increasing intensity after the baseline period with the traditional Mediterranean diet and after the period with the vegan version of the Mediterranean diet. Data are mean ± SD for 14 participants.

Figure 3 illustrates the impact of the traditional Mediterranean diet compared to the vegan Mediterranean diet on PFO and Fatmax during exercise. The diet did not affect either MFO (traditional Mediterranean diet: 0.323 g/min (0.153) and vegan Mediterranean diet: 0.347 g/min (0.l47); *p* = 0.678) or Fatmax (traditional Mediterranean diet: 40.51%VO_2peak_ (7.30) and vegan Mediterranean diet: 40.51%VO_2peak_ (10.71); *p* = 1.000).

**Figure 4:**
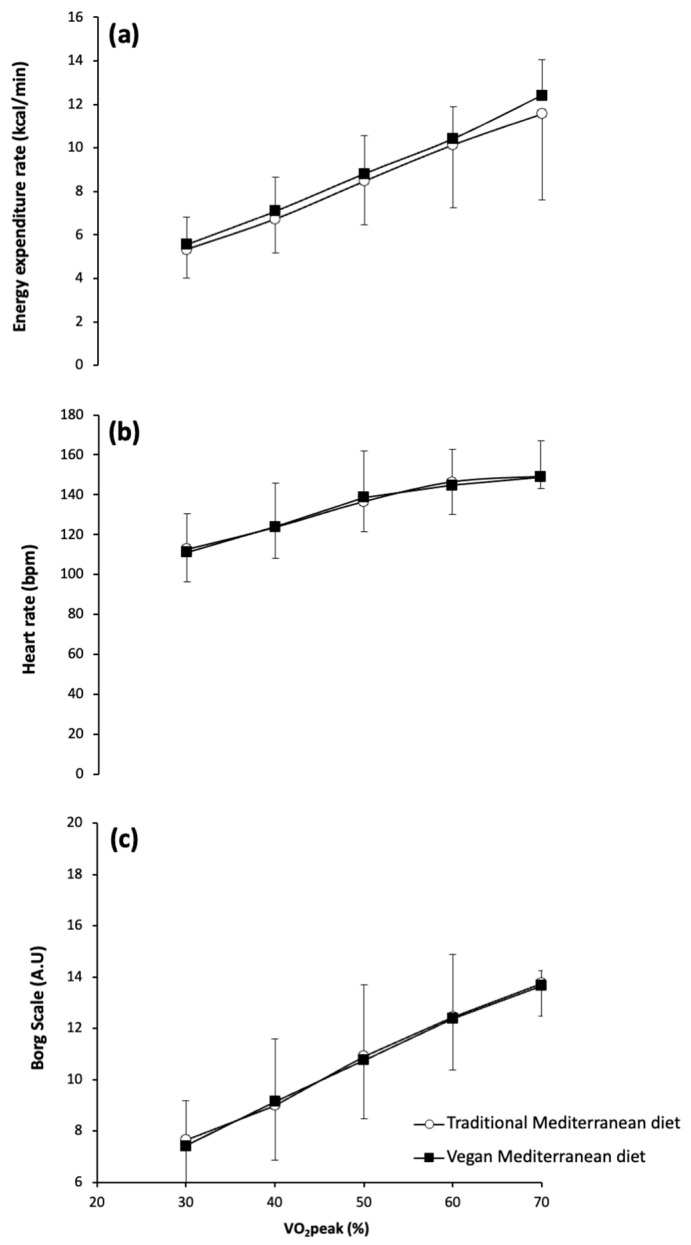
Energy expenditure rate (**a**), heart rate (**b**), and rating of perceived exertion measured with the 6–20-point Borg’s Scale (**c**) during exercise of increasing intensity after the baseline period with the traditional Mediterranean diet and after the period with the vegan version of the Mediterranean diet. Data are mean ± SD for 14 participants.

Figure 4 shows energy expenditure rate, heart rate, and the rating of perceived exertion across increasing exercise intensities for the traditional Mediterranean diet and vegan Mediterranean diet. No significant main effects were observed for diet (*F* = 0.069; *p* = 0.803) or for the diet × exercise intensity interaction (*F* = 1.355; *p* = 0.404), although exercise intensity produced a main effect on energy expenditure (*F* = 49.231; *p* = 0.005). A significant main effect of exercise intensity was also found for heart rate (*F* = 34.757; *p* = 0.008), while the main effects of diet (*F* = 6.335; *p* = 0.280) and the diet × intensity interaction (*F* = 1.704; *p* = 0.336) were not significant. Similarly, no significant main effects were detected for diet (*F* = 0.725; *p* = 0.433) or for diet × intensity interaction (*F* = 0.249; *p* = 0.858). However, exercise intensity had a significant main effect on the rating of perceived exertion measured using the Borg scale (*F* = 108.161; *p* < 0.001). Despite the significant main effect of exercise intensity across all variables, the posthoc analysis did not reveal any differences between the traditional and vegan Mediterranean diets at any intensity level for energy expenditure rate, heart rate, and the rating of perceived exertion.

With the exception of one participant who was excluded from the final analysis due to non-adherence to the guidelines of the traditional Mediterranean diet, all remaining participants successfully adhered to their assigned dietary interventions. No adverse effects or difficulties were reported, including common symptoms potentially associated with dietary changes—such as gastrointestinal discomfort; bloating; or other signs of digestive distress—particularly those often linked to increased fiber intake in vegan diets.

## 4. Discussion

The main objective of this study was to investigate the consequences of a controlled, isocaloric transition from a traditional Mediterranean diet to a vegan Mediterranean diet on substrate oxidation during exercise in healthy, physically active individuals. This investigation is relevant because previous studies have shown that the amount of dietary fat intake may influence substrate utilization during exercise, but there is limited information on how changing the type of fat consumed, particularly when transitioning to a vegan diet, affects this process. This is especially important when considering a shift from the traditional Mediterranean diet, which is already regarded as a healthy dietary pattern. Interestingly, while the traditional Mediterranean diet used in this study included both saturated and unsaturated fats (25.2 vs. 74.8%, respectively), derived from small amounts of animal sources such as fish, eggs, meat, and dairy products, the vegan adaptation of this dietary pattern increased the proportion of unsaturated fats up to 86.4% of the total amount of fat as a result of the removal of animal-based foods. Despite the dietary change, the main findings of this study indicate that transitioning from a traditional to a vegan Mediterranean diet was not associated with any change in fat or carbohydrate oxidation rates, peak fat oxidation, or Fatmax during exercise. From a practical perspective, these findings suggest that physically active individuals can adopt a vegan Mediterranean diet without compromising fat utilization during exercise while potentially gaining additional benefits in metabolic health, such as improved lipid profiles and reduced blood pressure, as previously reported in the initial OMNIVEG study [23].

Previous evidence indicates that dietary carbohydrate and fat intake are key modulators of PFO, with higher carbohydrate availability tending to suppress fat oxidation, whereas diets rich in fats may increase PFO during exercise [37,38]. In the present study, the carbohydrate and fat intakes (both in g/day and as a percentage of the total energy intake, Table 1) for the diet transition were tightly matched across both conditions, which may partly explain the absence of significant differences in substrate oxidation and energy expenditure during exercise. Still, compared to the traditional Mediterranean diet, the vegan Mediterranean diet provided an increased dietary fiber while slightly reducing protein intake, which is habitual when transitioning to a vegan diet [39,40]. Dietary fiber plays a crucial role in modulating gut microbiota composition and enhancing the production of short-chain fatty acids (SCFAs), such as acetate, propionate, and butyrate, which may influence lipid metabolism and insulin sensitivity [41,42]. SCFAs have been shown to increase mitochondrial efficiency and fat oxidation in both preclinical and clinical settings. However, the short duration of the intervention in the current study may have been insufficient to induce measurable changes in SCFA production or microbiota profiles, which could explain the lack of observable metabolic consequences despite significant differences in fiber intake.

Total fat intake, but also fat type, may modify the reliance on fat as a fuel for the working muscle during exercise. For example, diets with higher proportions of unsaturated fats, such as monounsaturated and polyunsaturated fatty acids, are often associated with higher fat oxidation during exercise [43]. However, the current study found that transitioning from a traditional Mediterranean diet to a vegan Mediterranean diet, which was characterized by a higher proportion of unsaturated fats, did not significantly modify fat or carbohydrate oxidation rates during exercise. This finding aligns with previous research by Boss et al. [27], which demonstrated that supplementing an endurance training regimen with a diet enriched in unsaturated fatty acids did not significantly augment fat oxidation compared to a control diet. These results suggest that, in the context of short-term dietary interventions, increasing unsaturated fat intake without changing the overall proportion of dietary fat relative to total energy intake may not modify fat oxidation during exercise. From a practical standpoint, this is a positive finding, as it means that the transition from a traditional to a vegan Mediterranean diet can be implemented without modifying total macronutrient intake, as the Mediterranean diet is already predominantly plant-based and contains only modest amounts of animal-derived fats. Additionally, this change will render a healthier diet pattern with less saturated fats but without impacting substrate oxidized during exercise.

The strengths of the present study include its controlled crossover design, individualized dietary planning, and comprehensive assessment of physiological parameters. However, it possesses several limitations that should be considered to understand the scope of the findings. The order of the dietary interventions employed in this study was not randomized, which may have introduced a potential order effect. We deliberately chose not to randomize the dietary sequence because all participants were Spanish individuals with a strong cultural and dietary familiarity with the traditional Mediterranean diet. Beginning with this familiar pattern helped ensure dietary compliance and reduced the likelihood of early dropout. More importantly, randomizing the order could have introduced carryover effects, particularly in participants who started with the vegan Mediterranean diet, potentially confounding the results. As such, the findings of this study are specifically applicable to individuals transitioning from a traditional to a vegan Mediterranean diet, and we cannot extrapolate these results to individuals making the reverse transition. The experiment had a short intervention period (i.e., 28 days after the transition) with a relatively small sample size, which may not fully capture the long-term impact of the dietary transition on substrate metabolism. Additionally, the use of self-reported dietary adherence, despite rigorous monitoring, introduces potential bias regarding the accuracy of adherence. Another important limitation is that the study was conducted exclusively in healthy, physically active males. This decision was made to reduce biological variability and enhance the internal validity of the findings, as sex-based differences in substrate metabolism are well documented [44]. To ensure valid comparisons between dietary phases in female participants, testing would need to be conducted during the same phase of the menstrual cycle (e.g., follicular or luteal; [45]). However, we were unable to implement a protocol to accurately determine or control for menstrual cycle phase. This restricts the generalizability of the findings, as we cannot ascertain whether similar results would be observed in female participants. Women are known to rely more on fat as a substrate during submaximal exercise compared to men [46,47], potentially leading to different physiological responses to dietary changes. Furthermore, the findings may not apply to high-performance or elite athletes, who typically exhibit higher PFO values and reach Fatmax at greater relative exercise intensities [48]. In such individuals, even subtle changes in dietary fat quality, such as a shift toward higher unsaturated fat intake, could produce more pronounced consequences on fat utilization due to greater metabolic demands and training-induced adaptations [49]. Finally, while this study provided detailed insights into substrate oxidation and related physiological responses, it did not assess endurance performance directly through a functional outcome, such as a time-to-exhaustion test or time trial. As a result, it remains unclear whether the observed similarities in fat oxidation rates between diets translate into equivalent outcomes on actual endurance capacity or performance outcomes.

## 5. Conclusions

In summary, switching from a traditional Mediterranean diet to a vegan Mediterranean diet did not affect peak fat oxidation, exercise intensity at peak fat oxidation, or other metabolic and perceptual responses during exercise in healthy, active men. These findings suggest that physically active individuals can adopt a vegan version of the Mediterranean diet by eliminating the small amounts of animal products typically included without compromising fat utilization during submaximal aerobic exercise. Importantly, the vegan Mediterranean diet may provide additional metabolic advantages related to cardiovascular and metabolic health, as discussed in previous research. Transitioning between these two dietary patterns is relatively straightforward, as the traditional Mediterranean diet is already plant-forward and minimally reliant on animal products. The increasing availability of plant-based food products with high protein content—such as tofu; tempeh; seitan; and fortified plant-based dairy alternatives—makes the substitution of animal-derived proteins more accessible. Similarly, replacing animal fats with additional olive oil or other plant-based fats is feasible and consistent with the core principles of the Mediterranean dietary pattern. These findings support the vegan Mediterranean diet as a practical, metabolically beneficial dietary approach for physically active individuals. Future research should explore the long-term physiological adaptations to plant-based Mediterranean diets, particularly in larger, more diverse populations and among athletes with higher metabolic demands.

## Figures and Tables

**Table 1:** Dietary characteristics of the traditional and vegan versions of the Mediterranean diet.

	Traditional Mediterranean Diet	Vegan Mediterranean Diet	*p*-Value	ES
Energy, kcal/day	2599.6 (180.8)	2634.9 (148.3)	0.14	0.25
CHO, g/day	311.3 (29.7)	320.8 (52.4)	0.39	0.22
CHO, % of energy	47.9 (3.3)	48.9 (5.1)	0.17	0.23
Animal CHO, % of CHO	6.4 (2.3)	0.0 (0.0)	<0.01	2.74
Dietary fibre, g/day	30.7 (2.8)	41.1 (4.9)	<0.01	2.61
Protein, g/day	120.2 (5.1)	115.3 (4.8)	<0.01	0.99
Protein, % of energy	18.5 (1.5)	17.5 (0.9)	<0.01	0.81
Animal protein, % of protein	62.9 (8.2)	0.0 (0.0)	<0.01	10.8
Fat, g/day	97.0 (17.8)	99.0 (13.2)	0.62	0.13
Fat, % of energy	33.6 (5.2)	33.8 (2.1)	0.90	0.05
Animal fat, % of fat	21.0 (6.0)	0.0 (0.0)	<0.01	4.95
Saturated fat, % fat	25.2 (6.8)	13.6 (4.4)	<0.01	2.03
Unsaturated fat, % fat	74.8 (9.3)	86.4 (5.9)	<0.01	1.49

Data are presented as mean (standard deviation). CHO = Carbohydrates, ES = Effect size. The proportion of animal-derived macronutrients was calculated for each diet. For example, animal-derived carbohydrates (e.g., lactose from dairy products), proteins (e.g., fish, meat, and poultry), and fats (e.g., from fish, meat, and poultry) had a low-to-moderate proportion of the total macronutrient content in the traditional Mediterranean diet but were absent in the vegan Mediterranean diet. To assess the impact of the transition from one diet to the other, participants underwent a Fatmax test at the conclusion of each dietary phase. This test, conducted on a cycle ergometer, was used to determine peak fat oxidation (PFO) and the exercise intensity at which PFO occurred, referred to as Fatmax, expressed as a percentage of VO_2peak_. All exercise testing was conducted under controlled environmental conditions, with standardized temperature and humidity levels to eliminate external influences on fat oxidation [29]. Additionally, testing sessions were scheduled in the morning to minimize the potential impact of circadian variation on metabolic responses. The impact of this dietary transition on blood lipid profiles, anthropometric variables, and blood pressure has been reported elsewhere [23].

## Data Availability

The raw data supporting the conclusions of this article will be made available by the authors on request.
